# Nuclear Receptor Unfulfilled Regulates Axonal Guidance and Cell Identity of *Drosophila* Mushroom Body Neurons

**DOI:** 10.1371/journal.pone.0008392

**Published:** 2009-12-22

**Authors:** Suewei Lin, Yaling Huang, Tzumin Lee

**Affiliations:** 1 Department of Neurobiology, University of Massachusetts Medical School, Worcester, Massachusetts, United States of America; 2 Janelia Farm Research Campus, Howard Hughes Medical Institute, Ashburn, Virginia, United States of America; Columbia University, United States of America

## Abstract

Nuclear receptors (NRs) comprise a family of ligand-regulated transcription factors that control diverse critical biological processes including various aspects of brain development. Eighteen NR genes exist in the *Drosophila* genome. To explore their roles in brain development, we knocked down individual NRs through the development of the mushroom bodies (MBs) by targeted RNAi. Besides recapitulating the known MB phenotypes for three NRs, we found that *unfulfilled* (*unf*), an ortholog of human *photoreceptor specific nuclear receptor* (*PNR*), regulates axonal morphogenesis and neuronal subtype identity. The adult MBs develop through remodeling of γ neurons plus de-novo elaboration of both α′/β′ and α/β neurons. Notably, *unf* is largely dispensable for the initial elaboration of γ neurons, but plays an essential role in their re-extension of axons after pruning during early metamorphosis. The subsequently derived MB neuron types also require *unf* for extension of axons beyond the terminus of the pruned bundle. Tracing single axons revealed misrouting rather than simple truncation. Further, silencing *unf* in single-cell clones elicited misguidance of axons in otherwise unperturbed MBs. Such axon guidance defects may occur as MB neurons partially lose their subtype identity, as evidenced by suppression of various MB subtype markers in *unf* knockdown MBs. In sum, *unf* governs axonal morphogenesis of multiple MB neuron types, possibly through regulating neuronal subtype identity.

## Introduction

The brain consists of neurons that are wired in specific patterns, and establishing a complex brain involves multiple tightly regulated developmental processes. In *Drosophila*, it starts with birth of neuroblasts (Nbs) with specific fates that are largely acquired through spatial patterning [1]. The Nbs then proliferate to produce multiple neuron types often in an invariant sequence [2], [3]. Post-mitotic neurons subsequently undergo extensive morphogenesis and some neurons remodel to form the circuitry [4], [5]. Although these basic processes are known, the detailed mechanisms that govern each step of brain development remain only partially resolved. Identifying more genes required for the various aspects of brain development is essential for elucidating further how the complex brain develops.

Nuclear receptors (NRs) are ligand-regulated transcription factors that play important roles in key metabolic and developmental pathways, including lipid and glucose homeostasis, aging, and cell fate determination [6], [7], [8], [9], [10], [11], [12], [13], [14] [15] [16]. NRs have also been shown to govern diverse aspects of neural development, such as the maintenance of neuronal precursors [17], [18], [19], neuronal cell death [20], axon guidance [21], [22], [23] and neuronal remodeling [24], [25]. Furthermore, mutations in NRs have been implicated in several neurodegenerative diseases [11], [15], [26], [27], [28], [29], [30], [31], [32]. Determining the functions of NRs will provide ample opportunities for better understanding brain development and neuron degeneration.

NR genes are highly conserved across the animal kingdom. The 48 NR genes identified in mammals can be categorized into six subfamilies according to their protein structure similarity [33]. The neural functions of most NRs have not been explored at all. Compared to the 48 NR genes in mammals, the *Drosophila* genome contains only 18 NR genes, though all six NR subfamilies are represented [33]. The smaller number of NR genes makes it easy to survey NR functions in brain development. Four fly NRs have been shown to regulate various aspects of neural development. *Seven-up* (*svp*), which encodes a homolog of human COUP-TF orphan NR, represses the expression of a temporal identity gene *hunchback* (*hb*) in embryonic neuroblasts after the first mitosis to ensure the subsequently produced neurons acquire proper cell fates [8]. Moreover, in the late larval stage, a burst expression of *svp* is required for neuroblasts to exist the cell cycle or undergo apoptosis [34]. *Tailless* (*tll*), the *Drosophila* homolog of human *Tlx* orphan NR, is robustly expressed in the larval brain in certain neuroblasts and ganglion mother cells (GMCs) to promote cell cycling and prevent apoptosis [17]. *Ecdysone receptor* (*EcR*) and its heterodimeric partner *ultraspiracle* (*usp*) work together to regulate axonal and dendritic remodeling during metamorphosis [24], [25], [35], [36], [37]. EcR have also been shown to regulate programmed neuronal cell death [20], [38] and secondary arbors of adult-specific neurons [39]. However, the functions of the remaining 14 NRs in neural development have not been studied.

The *Drosophila* mushroom body (MB), the olfactory learning/memory center, has been shown as an excellent model to study gene functions in neural development (e.g. [24], [40], [41], [42]), and thus could also be good for studying NR functions. The adult MB consists of three major types of neurons- γ, α′/β′ and α/β [43]. These neuron types are specified according to their date of birth by temporal identity factors, such as *chinmo* [44]. The γ neurons are born before the mid-3rd instar larval stage [around 4 days after larval hatching (ALH)]; their axons initially form bifurcated branches in the larval brain. Later, these axons are pruned through fragmentation and glia engulfment at early pupal stage, followed by the re-extension of adult-specific axons that only elaborate horizontally toward the midline of the adult brain [43], [45]. The α′/β′ neurons are born between the mid-3rd instar and puparium formation, and α/β neurons are born at the pupal stage. Both α/β and α′/β′ axons form bifurcated branches without remodeling during development, and thus may only be functional in the adult brain [43]. As to roles of NRs in MB development, EcR, USP and TLL have been shown playing important roles in regulating MB remodeling and neurogenesis, respectively (see below) [17], [24].

In this paper, we systematically silenced each of the 18 *Drosophila* NR genes in MBs using miRNA-based RNA interference [46], [47]. In addition to *EcR*, *usp*, and *tll*, we isolated *unfulfilled* (*unf*), the fly NR2E3 subfamily ortholog of *C. elegans fax-1* and human *photoreceptor-specific nuclear receptor* (*PNR*) [48], [49]. *unf* has a novel function in MB development, and *unf* knockdown caused a severe MB lobe extension defect. Further analysis revealed that *unf* was required for proper axonal guidance of all three major types of the MB neurons. Without *unf*, the MB axons were misguided and wandered around at the end of peduncle. Interestingly, *unf* was mainly required for adult-specific axonogenesis. In later-born adult-specific α′/β′ and α/β neurons, *unf* played a critical role in initial neurite extension; in contrast, for early-born γ neurons, *unf* was largely dispensable to the establishment of larval projections, but absolutely required for their re-elaboration of axons during early metamorphosis.

We also found that the expression of Trio, a guanine nucleotide exchange factor important for the MB axonal guidance [50], as well as several subtype-specific markers were significantly reduced in the *unf* knockdown adult MB. This result implies that *unf* is required for MB neurons to acquire correct gene expression profile (i.e. cell identity) and that improper expression of multiple guidance molecules resulting from the loss of neuron identities might underlie the axonal defects induced by *unf* knockdown.

## Results

### Silencing Individual NRs by miRNA Unveils a Role of Nuclear Receptor *unf* in MB Development

To study NR functions in fly brain development, we generated *UAS-miRNA* transgenic fly lines against each of the 18 nuclear receptor (NR) genes so far identified in the fly genome. We drove the miRNA transgenes using *GAL4-OK107* [51] to knock down individual NRs through development of the MBs. We detected MB abnormalities when the miRNAs against *ecdysone receptor* (*EcR*), *ultraspiracle* (*usp*), *tailless* (*tll*), *ftz transcription factor 1* (*ftz-f1*) or *unfulfilled* (*unf*) were induced (Table S1).

**Figure 1:**
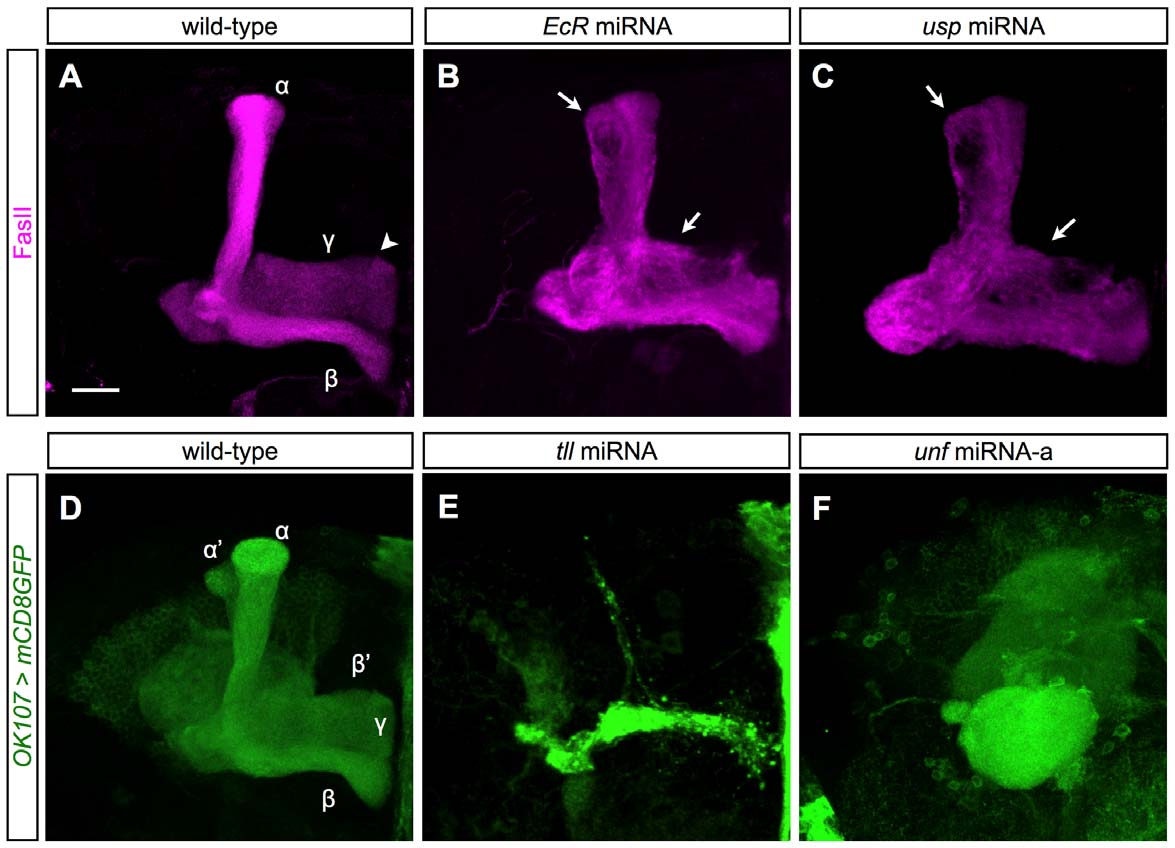
*unf* is required for the proper axonogenesis of the mushroom body neurons. (A–C) Anti-FasII antibody staining (magenta) revealed the γ and α/β lobe morphology of wild-type (A), *EcR-* (B) and *usp-* (C) knockdown adult MBs. The knockdown was induced by *GAL4-OK107* dependent induction of miRNAs. Note the *EcR-* and *usp-*knockdown MBs contained unpruned γ processes (arrows in [B] and [C]) and lacked a normal-looking γ lobe (e.g. arrowhead in [A]). Scale bar: 20 µm. (D–F) Adult wild-type (D), *tll*- (E) and *unf-* (F) knockdown MB, labeled by *GAL4-OK107* (green). The knockdown was induced by *GAL4-OK107* dependent induction of miRNAs. Note the *tll-*knockdown MB contained only few neurons (E), and the axon lobes of *unf-*knockdown MB failed to extend along the correct paths (F).

*EcR* and its heterodimeric partner *usp* have been shown to regulate the axonal pruning of MB γ neurons [24], and *tll* was recently demonstrated to promote the efficient proliferation of the MB neuroblasts and *ganglion* mother cells [17]. Our miRNA screening results were consistent with the previous studies. Knocking down *EcR* or *usp* blocked pruning of MB γ neurons, which maintained larval-type projections in the adult brain (Fig. 1A–C). In contrast, silencing *tll* affected MB proliferation, resulting in a tiny MB purely consisting of early-type MB neurons (Fig. 1D,E). This recapitulates the *tll* mutant MB phenotype [17]. The consistency between the miRNA results and published data suggests that our miRNA approach worked efficiently in knocking down the endogenous nuclear receptors.

In addition to the nuclear receptors known to be required for MB development, we found that silencing *ftz-f1* and *unf* caused abnormal MB morphologies. In the MB, distinct sets of MB axon bundles show different levels of FasII expression [52]; but silencing *ftz-f1* elicited ectopic FasII-positive bundles in about 30% of MBs (Table S1; data not shown). Ectopic FasII bundles could result from axon guidance defects, misregulation of cell fate, or incomplete γ axon pruning. Since the phenotype was subtle and the penetrance was low, we did not pursue analysis of *ftz-f1* in this study. In contrast, silencing *unf* caused a much stronger MB phenotype (Table S1; Fig. 1F); the five MB-characteristic axon lobes were missing in all *unf* knockdown MBs (Fig. 1F). While most axons might extend across the brain through the peduncle, they failed to form the lobes that normally project on the anterior surface of the brain hemisphere. Instead, the stereotyped MB lobes were replaced with a ball-like structure around the end of peduncle (Fig. 1F). The MBs were otherwise grossly normal, consisting of many cell bodies residing over the dendrite-based calyx that sometimes appears slightly larger than normal (data not shown).

**Figure 2:**
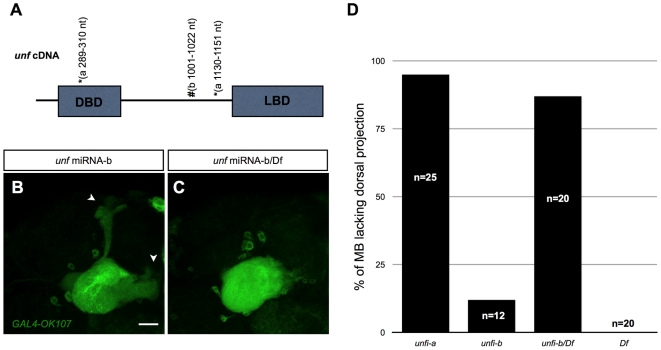
The MB lobe phenotype was caused by specific knockdown of *unf* by miRNA. (A) Illustration of the *unf* cDNA that contains a 5′ region encoding a putative DNA binding domain (DBD) and a 3′ region encoding a putative ligand binding domain (LBD). *The miRNA target sites for *unf* miRNA-a. #The miRNA target site for *unf* miRNA-b. (B–C) *unf* in MB was knocked down by the *GAL4-OK107* induced *unf* miRNA-b in a wild-type fly (B), or a fly heterozygous for a deficiency *Df(2R)ED2426* that covers *unf* gene (C). Note some MB axons can still project to their correct positions when the *unf* miRNA-b was induced in the wild-type background (indicated by arrowheads in [B]). Scale bar: 20 µm. (D) Statistic results of the percentage of MB lacking dorsal projection in flies heterozygous for *Df(2R)ED2426* (*Df*), or in flies with *OK107-*dependent induction of *unf* miRNA-a(*unfi-a*), *unf* miRNA-b (*unfi-b*), or *unf* miRNA-b plus one allele of *Df(2R)ED2426* (*unfi-b/Df*).

The *unf* miRNA-a used in the initial screening targets two sites on the *unf* coding sequence, one at 289–310 nt and the other at 1130–1151 nt (Fig. 2A). To ascribe the above phenotype to loss of *unf*, we first learned that the induction of this miRNA by *GAL4-OK107* did effectively deplete *unf* protein in MB neurons (see below). We then tried to rule out off-target effects by generating *unf* miRNA-b to target an independent site at 1001–1022 nt (Fig. 2A). *GAL4-OK107*-dependent induction of *unf* miRNA-b also led to a similar MB lobe extension defect, though the phenotype was weaker with some axons fully extended (Fig. 2B). To confirm that these phenotypes are indeed due to *unf* knockdown, we induced *unf* miRNA-b in flies heterozygous for a deficiency [*Df(2R)ED2426*] that covers the *unf* locus, and found that the MB axonal lobe defect became as severe as that caused by *unf* miRNA-a (Fig. 2C,D). Notably, when the *unf* miRNA-a was induced in the deficiency heterozygous background, the lobe phenotype was not enhanced (data not shown), suggesting that the abnormality caused by *unf* miRNA-a was a very strong *unf* loss-of-function phenotype. Taken together, these results show that *unf* is essential for the extension of the MB axonal lobes.

### *unf* Is Mainly Required for the Formation of Adult-Specific MB Lobes

**Figure 3:**
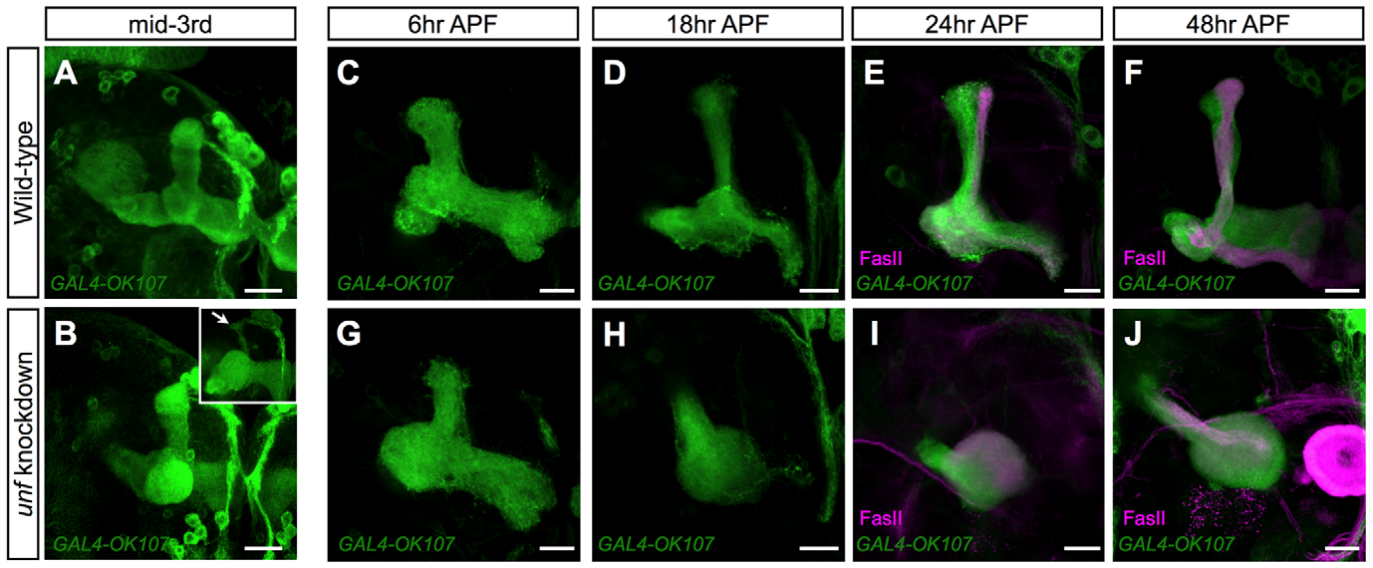
*unf* is mainly required for the formation of adult-specific MB lobes. Wild-type (A, C–F) and *unf* knockdown (B, G–J) MB at the indicated developmental stages were labeled by *GAL4-OK107* (A–J). The insect in [B] is an example of a small portion (12%; n = 75) of the *unf* -knockdown larval MB that displayed a thinner dorsal lobe phenotype. Brains in [E], [F], [I] and [J] were counterstained with anti-FasII antibody (magenta). *unf* was knocked down by *GAL4-OK107* induced *unf* miRNA-a. Scale bars: 20 µm.

To determine the pathological mechanisms underlying the lobe defects, we followed *unf* knockdown MBs through development. We found that most *unf* knockdown MBs (88%, n = 75) were grossly normal at mid-3rd instar larval stage when the MB primarily consists of γ neurons (Fig. 3A,B), although 12% of *unf* knockdown MBs had thinner dorsal lobes (n = 75; inset in Fig. 3B). This is in great contrast to the 100% lobe extension defect of *unf* knockdown adult MBs (e.g. Fig. 1F). This result was not due to the insufficient RNAi knockdown in the early larval stage, because *GAL4-OK107* induced *unf* miRNA can knockdown UNF protein completely within 6 hr ALH (See below). The observations suggest that *unf* is mainly involved in the formation of the adult MBs.

The adult MBs develop through remodeling of γ neurons plus de-novo elaboration of both α′/β′ and α/β neurons. These dynamic changes in the MB structure can be closely examined during early pupal development. In wild-type MBs, the bifurcated larval γ lobes were completely pruned around 18 hr APF, and at the same time the α′/β′ lobes that were initially wrapped by the larval γ lobes can be clearly seen (Fig. 3C,D). Around 24 hr APF when the adult-specific γ lobes started to extend, the nascent α/β axonal bundles derived from the pupal-born α/β neurons can be detected with anti-FasII mAb 1D4 (Fig. 3E). By 48 hr APF, the adult-specific γ lobe is fully extended and the α/β bundles become much thicker (Fig. 3F).

Examination of *unf* knockdown MBs through early pupal development revealed multiple abnormalities. First, mutant MBs at 6 hr APF displayed no obvious morphological defect (Fig. 3G). By contrast, at 18 hr APF when the larval γ lobes were largely pruned, there did not exist α′/β′ lobes (Fig. 3H). Second, no axon has extended beyond the peduncle end at 24 hr APF, indicating defects in both γ axon re-extension and α/β bundle formation (Fig. 3I). Third, by 48 hr APF, instead of seeing all five MB lobes, we detected a ball-like structure bulging around the terminus of the peduncle (Fig. 3J). It appears that MB axons were lost at the peduncle end. They elaborated locally, but failed to project into discrete domains based on their subtype identity. These observations reveal that 1) *unf* is largely dispensable for the initial larval-specific axonal morphogenesis of the γ neurons, but absolutely essential for the extension of the adult-specific γ axons during remodeling; 2) *unf* is necessary for the de-novo formation of α′/β′ and α/β lobes.

### *unf* Acts in Mature γ Neurons to Govern Axon Re-Extension, while Supporting Initial Axonal Morphogenesis in Later Types of MB Neurons

**Figure 4:**
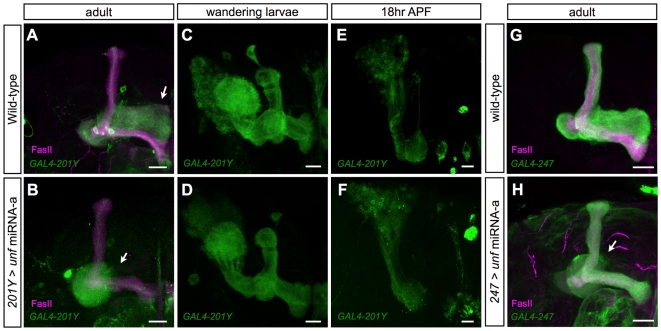
*unf* acts in mature γ neurons to govern axon re-extension. Wild-type (A,C,E,G) and *unf* knockdown (B,D,F,H) MB at the indicated developmental stages, labeled by *GAL4-201Y* (A–F) or *GAL4-MB247* (G,H). Brains in [A], [B], [G] and [H] were counterstained with anti-FasII antibody (magenta). *unf* was silenced by *GAL4-201Y* (B,D,F) or *GAL4-MB247* (H) induced *unf* miRNA-a. The arrows indicate the position of normal (A) and truncated γ lobes (B,H). Scale bars: 20 µm.

Given its pleiotropic functions in MB morphogenesis, we sought to determine the role of *unf* in γ neuron remodeling independent of its effects on other MB neuronal morphogenetic processes. We selectively knocked down *unf* in mature larval γ neurons using *GAL4-201Y* [53], resulting in missing adult γ lobes despite presence of other MB lobes (Fig. 4A,B). Phenotypic analysis through development revealed normal pruning of the larval-specific γ lobes (Fig. 4C–F) but no formation of the adult γ lobe (Fig. 4B). This result suggests that *unf* acts in mature γ neurons to promote axon re-extension during MB remodeling. In addition, lacking the γ lobe did not affect formation of MB α/β lobes (Fig. 4B).

**Figure 5:**
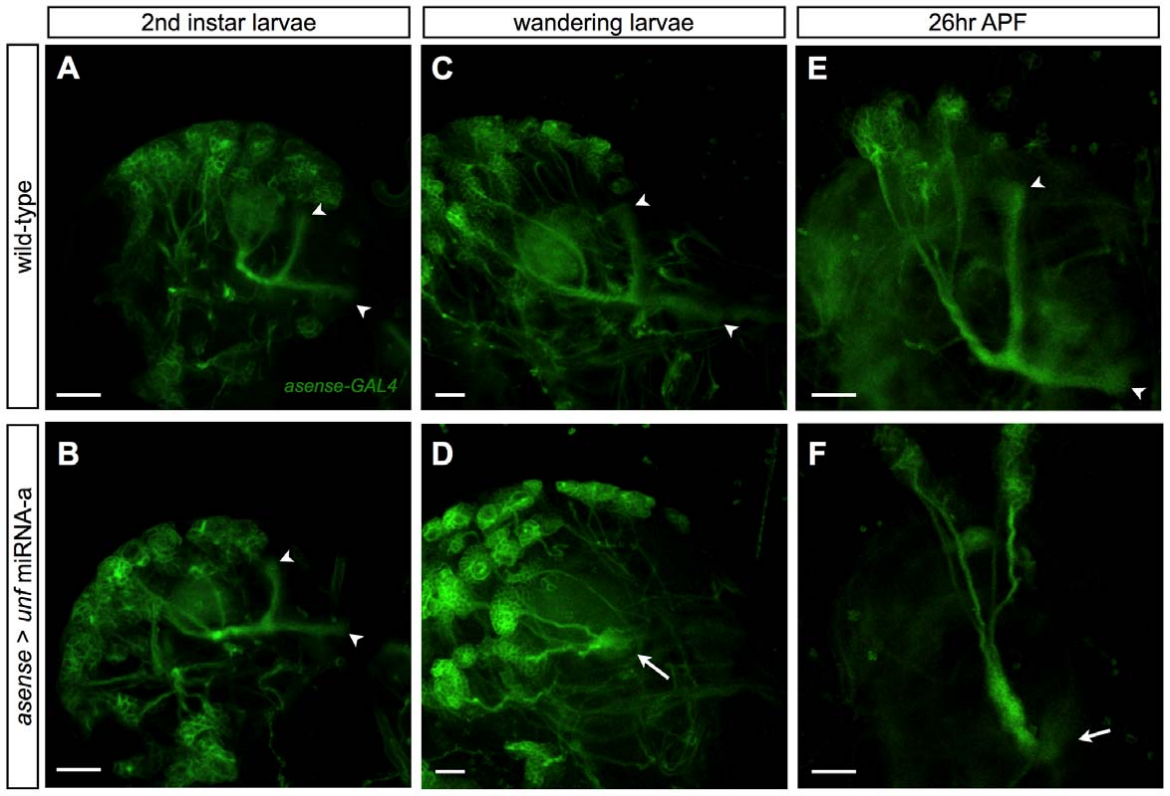
*unf* is required for the initial axonal morphogenesis in later types of MB neurons. Newly generated neurons of wild-type (A,C,E) and *unf* knockdown (B,D,F) MB labeled by *asense-GAL4* (green) at the indicated developmental stages. *unf* was knocked down by *asense-GAL4* dependent induction of *unf* miRNA-a. The arrowheads in [A]–[C] and [E] indicate the normal axonal bundles of the larval MB. The arrows in [D] and [F] indicate the axonal bundles that failed to extend beyond the MB peduncle. Scale bar: 20 µm.

Notably, the selective loss of the adult γ lobe was evident even when *unf* was silenced in most, if not all, post-mitotic MB neurons via induction of *unf* miRNA-a using *GAL4-MB247* (Fig. 4G,H). *GAL4-MB247* drives UAS-transgene expression in MB neurons that have undergone extensive morphogenesis [44]. This again indicates the requirement for *unf* in mature γ neurons, and further suggests that α′/β′ and α/β neurons need *unf* during their initial development. Consistent with these notions, silencing of *unf* in newborn neurons using *asense-GAL4* [44] did not affect larval γ axon projections (Fig. 5A,B) but drastically arrested the nascent α′/β′ (Fig. 5C,D) or α/β axon bundles (Fig. 5E,F) around the peduncle. Taken together, *unf* acts in mature γ neurons to govern axon re-extension while supporting axonal morphogenesis in newly derived α′/β′ and α/β neurons.

### Direct Involvement of *unf* in Later MB Morphogenetic Processes

The serially-derived MB neurons, made concurrently by four MB progenitors, have undergone morphogenesis in sequence. It has been shown that the projections of specific MBs could be affected by the trajectories of others [50]. Notably, all MB axons stalled around the peduncle end when *unf* was depleted through development of the MBs, raising the possibility that some later morphogenetic defects may occur as a consequence of earlier axons' failings. The above observation that the α/β lobes could form normally in the absence of the adult γ lobe (Fig. 4B,H) partially rules out such possibility. However, the α′/β′ lobes remained in those γ-lobe-missing MBs (Fig. 4B,H) and might play an essential role in guiding the later derived α/β axons at the peduncle end, a critical choice point to all MB axons.

**Figure 6:**
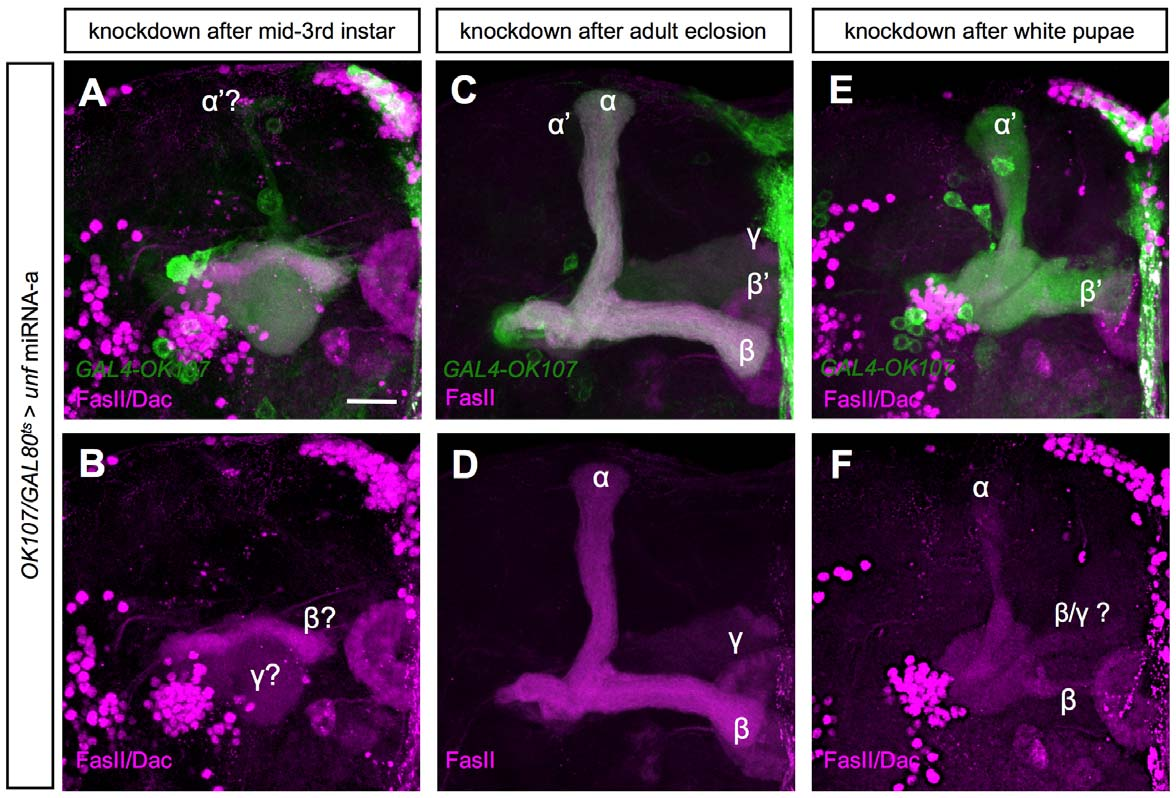
Direct involvement of *unf* in later MB morphogenetic processes. GAL80[ts] was used to control the timing of *GAL4-OK107* dependent induction of *unf* miRNA-a to knock down *unf* in MB after mid-3rd instar (A–B), after adult eclosion (C–D) or after puparium formation (E–F). MBs were labeled by *GAL4-OK107* (green; A,C,E), anti-FasII (magenta; A–F) and anti-Dac (magenta; A,B,E,F) immunostaining. Scale bar: 20 µm.

To demonstrate that *unf* is directly involved in the axonogenesis of α/β neurons, we sought to knock down *unf* after morphogenesis of γ and α′/β′ neurons to selectively deplete *unf* from newborn α/β neurons. We controlled the timing of targeted RNAi using a temperature-sensitive GAL4 repressor, GAL80[ts] [54]. Organisms with the genotype of *tubp-GAL80[ts],UAS-mCD8GFP/UAS-unf-miRNA-a;tubp-GAL80[ts]/+;GAL4-OK107/+* were initially cultured at a permissive temperature (18 degree Celsius) and shift to a restrictive temperature (29 degree Celsius) to induce the expression of *unf* miRNA-a at desired developmental stages. Besides labeling all five MB lobes by *GAL4-OK107*, we counterstained MB with anti-Trio antibody that strongly labels α/β lobes and weakly labels γ lobe [52] to make the five MB lobes distinguishable. Induction of *unf* RNAi from mid-3^rd^ instar and prior to birth of most α′/β′ neurons disrupted all MB lobes (Fig. 6A,B). In contrast, shifting the temperature after adult eclosion left the gross MB morphology intact (Fig. 6C,D). Intriguingly, when the temperature was shifted around puparium formation after the production of α′/β′ neurons, the α′/β′ lobes were grossly normal but the FasII-positive α/β lobes were malformed (Fig. 6E,F). These results demonstrate that the *unf* knockdown phenotype in α/β neurons was not due to the defects in α′/β′ lobes. Thus, *unf* directly governs axonal morphogenesis in all three major types of MB neurons.

### *unf* Regulates Axon Pathfinding of MB Neurons

**Figure 7:**
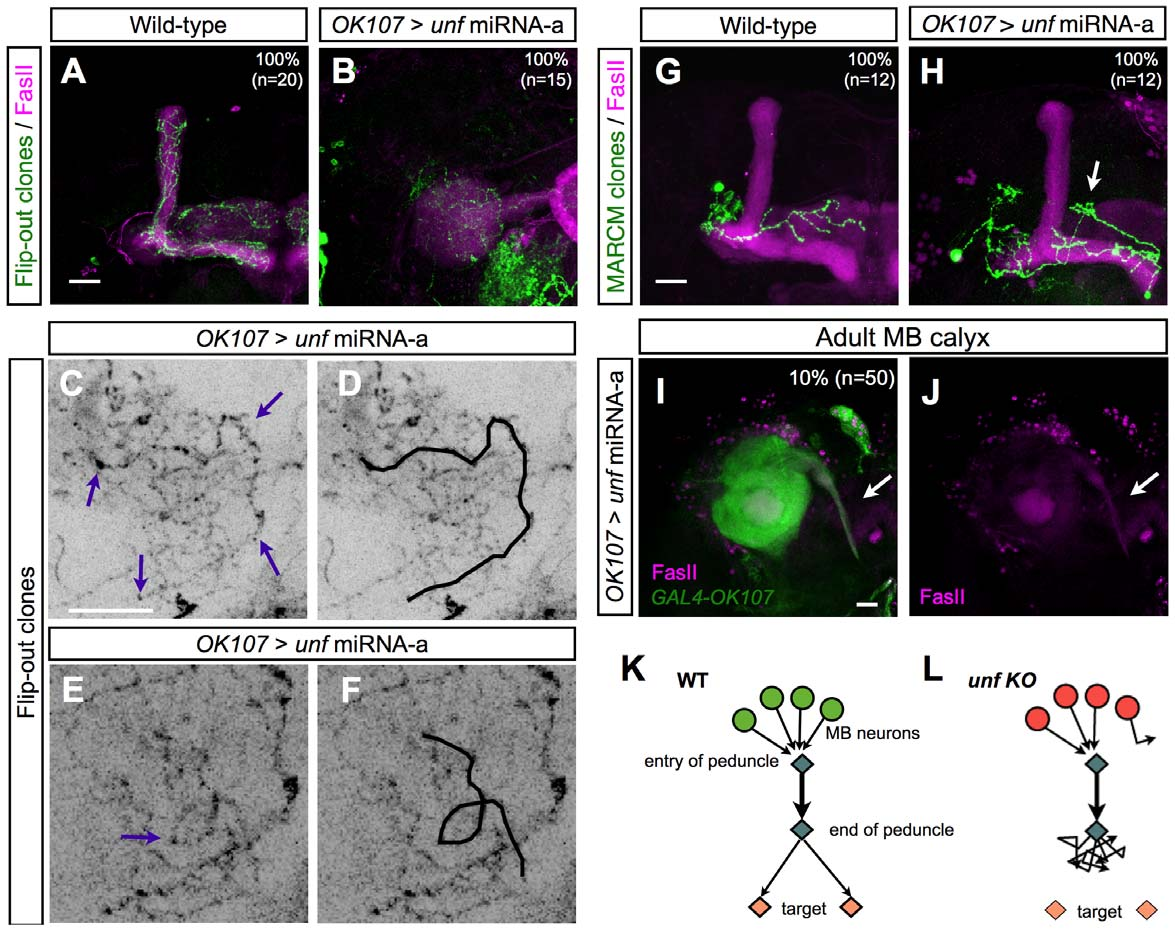
*unf* regulates axon pathfinding of MB neurons. (A–F) *GAL4-OK107* labeled flip-out clones (green in [A] and [B]; black in [C]–[F]) induced in a wild-type (A) or a *unf* knockdown (B–F) MB. Brains in [A] and [B] were co-labeled with anti-FasII Ab (magenta). [C]–[F] are high magnification views of some axons in the MB lobe in [B]. The arrows in [C] and [E] mark the axons with an unusual back turn(C), or even form a loop (E). [D] and [F] are duplicate images of [C] and [E] with the abnormal axonal paths highlighted by black lines for better visualization. Scale bars: 20 µm. (G–H) Wild-type (G) and *unf* knockdown (H) MARCM clones labeled by *GAL4-OK107* (green). *unf* was knocked down by *GAL4-OK107* induced *unf* miRNA-a. Brains were counterstained with anti-FasII Ab (magenta) to mark MB γ and α/β lobes. The arrow in [H] indicates a misrouted axon. Scale bar: 20 µm. (I–J) The calyx region of a *unf* knockdown adult MB labeled by *GAL4-OK107* (green; I) and anti-FasII immunostaining (magenta; I–J). *unf* was knockdown by *GAL4-OK107* induced *unf* miRNA-a. The arrows indicate a misrouted FasII-positive axonal bundle. Scale bar: 20 µm. (K–L) A illustration of the axonal projections in wild-type (K) and *unf* knockdown (L) MB.

MB axons uniformly stalled around the peduncle end, a common choice point to the migrating axons, raising the possibility that *unf* may regulate axon pathfinding rather than simply promoting axon extension. To elucidate the mechanism(s) underlying the axonal lobe defect of the *unf* knockdown MBs, we followed single axons to determine their trajectories in the lobe defective MBs. Single MB neurons were labeled using flip-out strategy [55], [56]. In wild-type MBs, the flip-out clones consistently projected their axons directly into the MB lobes (Fig. 7A). In contrast, the axonal processes of flip-out clones in the *unf* knockdown MBs wandered around in the ball-like truncated lobes (Fig. 7B). These wandering axons were apparently lost, and often made unusual back turns or loops (Fig. 7C–F). These phenomena ascribe the lobe defect of the *unf* knockdown MB to misrouting of axons.

We further determined if *unf* is cell-autonomously required in individual MB neurons for proper axon guidance, by knocking down *unf* in single MB neurons. We generated isolated single-cell clones of γ neurons by MARCM (Mosaic Analysis with a Repressible Cell Marker) [57]. Such uniquely labeled cells were the only MB neurons that lack the GAL4 repressor, GAL80, and thus actively expressed *unf* miRNA-a. This allowed us to knock down *unf* in single MB neurons within otherwise unperturbed MBs. Notably, the lone *unf* knockdown neurons exhibited abnormal axon trajectories in the grossly normal MBs (Fig. 7G,H). The misguided axons were not stalled at the peduncle end, possibly because their surrounding wild-type axons may somehow steer the *unf* knockdown axons into the lobes. Additional evidence for the involvement of *unf* in axon guidance came from the observation that about 10% of the strongest *unf* knockdown MBs showed ectopic FasII-positive axon bundles extending through the calyx into abnormal targets rather than migrating along the peduncle (Fig. 7I,J). Taken together, these observations suggest that *unf* acts cell-autonomously to regulate individual axons' pathfinding (Fig. 7K,L).

### *unf* Is Expressed in MB Neurons Continuously to Regulate Neuron Subtype Identity

**Figure 8:**
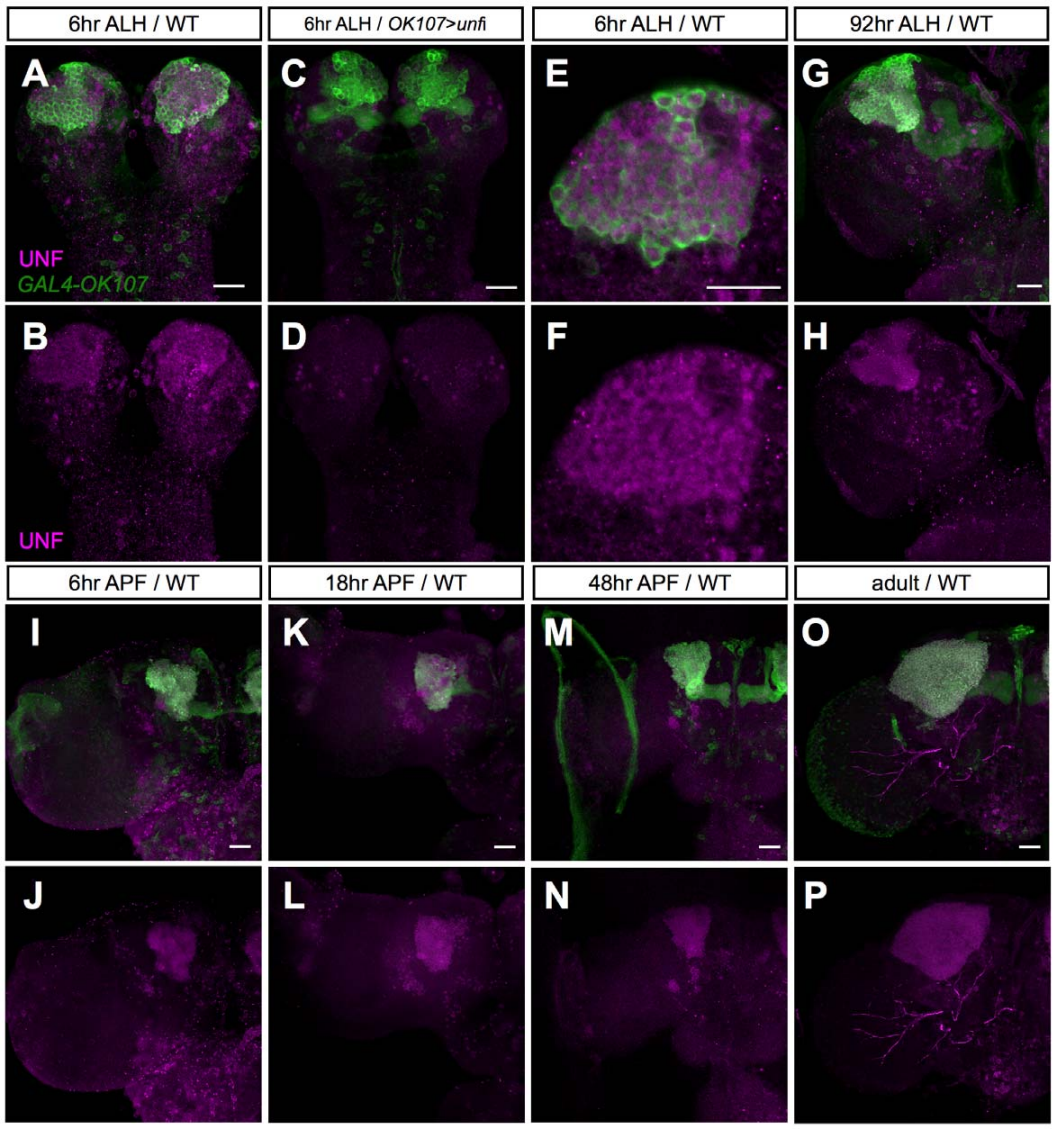
*unf* is continuously expressed in the MB neurons throughout development. Wild-type brains (A,B,E–P) at the indicated development stages and a 6 hr-old larval brain with *GAL4-OK107* dependent induction of *unf* miRNA-a (C–D) were labeled with *GAL4-OK107* (green; A, C, E, G, I, K, M and O) and UNF immunostaining (magenta; A–P). Scale bars: 20 µm.

In the MB γ neurons, *unf* is required in a stage-specific manner. Genes required for remodeling of MBs may dynamically express in response to ecdysone signaling. To determine the expression of *unf*, we generated a rabbit polyclonal antibody against a short peptide (DVTNDNEEPHA) characteristic of *unf*. The antibody recognized abundant *unf* expression in the MB neuronal cell bodies (Fig. 8). Depleting *unf* by targeted RNAi greatly suppressed the immunostaining signal (Fig. 8A–D), confirming the specificity of the antibody. Notably, *unf* is enriched in all MB neurons through different developmental stages (Fig. 8). No dynamic changes in its expression could be detected during MB remodeling, and high-level expression continues in adult MB neurons (Fig. 8). This expression pattern provides no clue as to why *unf* is essential for γ neuron remodeling but largely dispensable for their initial morphogenesis. However, its enrichment in the MBs strongly supports our observations that *unf* controls some MB-characteristic aspects of neural development.

**Figure 9:**
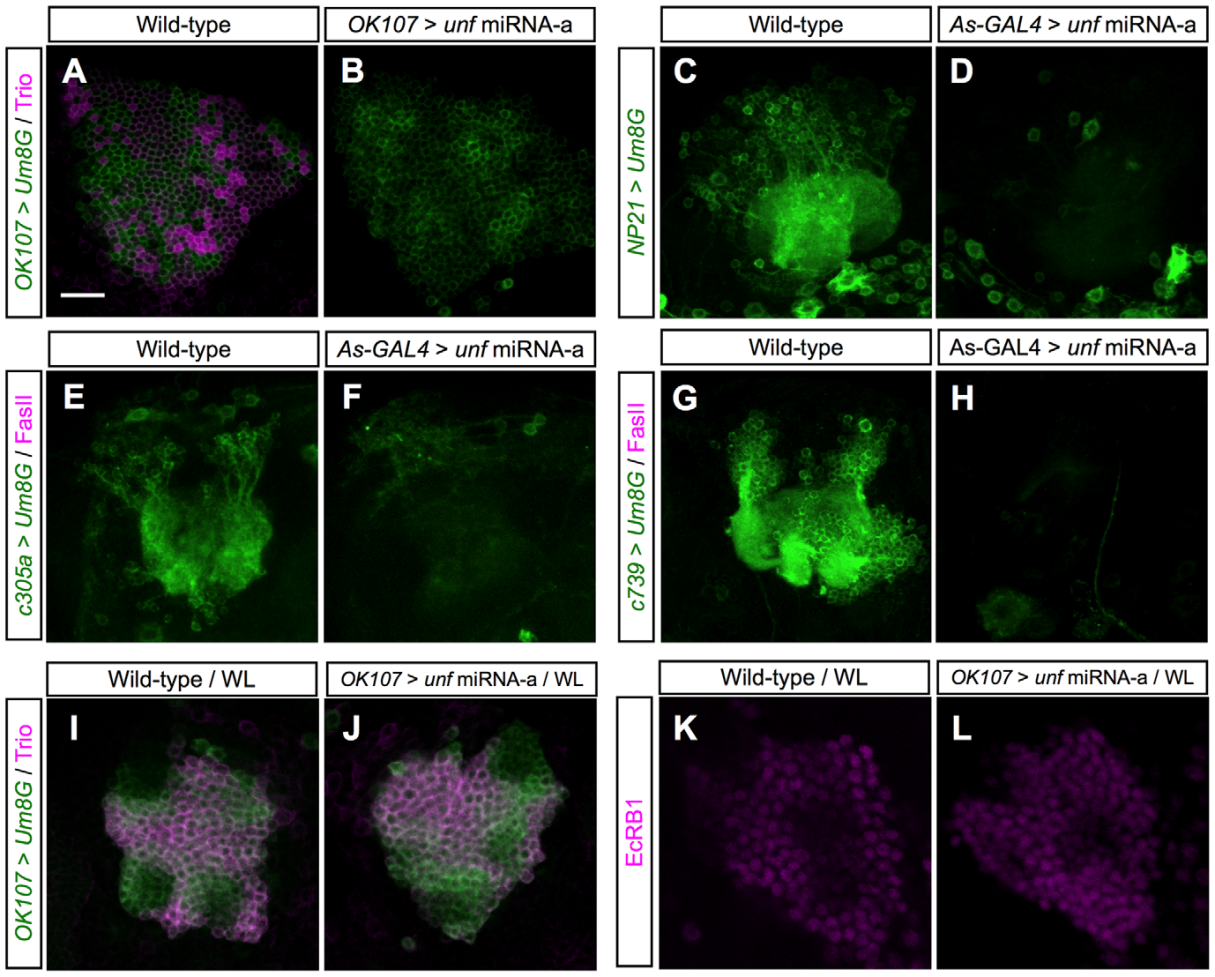
Subtype-specific markers were lost in *unf* knockdown adult MBs. The cell body region of wild-type (A, C, E, G, I and K) or *unf* knockdown (B, D, F, H, J and L) adult (A–H) or wandering larval (I–L) MBs labeled with various MB specific markers, including Trio (A, B, I and J; magenta), *GAL4-NP21* (C and D; green), *GAL4-c305a* (E and F; green), *GAL4-c739* (G and H; green) and EcR-B1 (K and L; magenta). Brains in [A], [B], [I] and [J] were co-labeled with *GAL4-OK107*. The *unf* miRNA knockdown in [A], [B] and [I]–[L] was induced by *GAL4-OK107*, and the *unf* knockdown in [C]–[H] was induced by *asense-GAL4* together with *GAL4-NP21* (C and D), *GAL4-c305a* (E and F), or *GAL4-c739* (G and H). Note the Trio expression was normal in *unf* knockdown larval MB cell bodies (J), but became undetectable in *unf* knockdown adult MB cell bodies (B). *Um8G*: *UAS-mCD8::GFP*. Scale bar: 20 µm.

Human *PNR* and *C. elegans fax-1*, the orthologs of fly *unf*, have been shown to regulate neuron identity [11], [22], [58], [59]. To investigate if *unf* controls MB neuron identity and subsequently governs the subtype-specific axon projections, we examined the neuronal cell fate in *unf* knockdown MBs. We achieved this by knocking down *unf* using *asenase-GAL4* in combination with different Pan-MB or MB subtype-specific markers, including several GAL4s. Given that *asense-GAL4* expresses in MB precursors and developing young MB neurons [44], it allows us to knock down *unf* throughout MB development; and because *ansense-GAL4* does not express in adult MB, it will not interfere with the expression of the other MB-specific GAL4s in the adult brain. None of the pan-MB markers, including *dachshund* (*dac*) [60], [61], *GAL4-OK107* [51] and *GAL4-MB247* [62], was affected by *unf* knockdown (data not shown), suggesting that the MB lineage identity was not regulated by *unf*. However, all the subtype-specific markers we examined lost their expression in the *unf* knockdown adult MBs. These include the γ and α′/β′-specific marker Trio in MB cell bodies (some Trio protein can be detected in MB lobes, which might be the residual product of earlier *trio* expression; see below) [50] (Fig. 9A,B), the γ-specific maker *GAL4-NP21* [63] (Fig. 9C,D), the α′/β′-specific marker *GAL4-c305a* [64] (Fig. 9E,F), the pioneer α/β-specific marker *GAL4-c708* [44](data not shown) and the α/β-specific marker *GAL4-c739* [53] (Fig. 9G,H). Thus, MB neurons require *unf* to acquire their subtype identity.

Notably, at the wandering larval stage, when the morphology of the *unf* knockdown γ neurons was mostly normal, the expression of the γ-specific genes such as *trio* or *EcR-B1* [24], [50] was unaffected (Fig. 9I–L). The loss of the γ-specific markers during metamorphosis suggests that there is a second wave of cell fate specification and/or consolidation occurring at the larva-to-pupa transition for the γ neurons to acquire their final fate; and *unf* is possibly required for this process. In sum, loss of neuron subtype identity may underlie misguidance of axons in *unf* knockdown MBs.

## Discussion

Silencing individual NRs through development of the MBs by transgenic miRNAs has allowed us to identify *unf* as another NR (in addition to *EcR*, *usp*, and *tll*) that regulates MB development. Previously, *unf* was shown to be essential for the wing expansion and fertility of adult flies, and abundantly expressed in the developing MBs [49]. Here we studied the function of *unf* in further detail and learned that *unf* acts in all three major types of MB neurons to promote proper neuron subtype identity and axon guidance. Comparable axon stalling defects of adult MB neurons, as well as missing larval MB dorsal axonal branches were observed in *unf* mutant organisms (Bates et al., submitted), validating our study of *unf*'s mechanism of action by targeted RNAi.

The involvement of *unf* in cell fate determination is evident by the loss of subtype-specific markers in the *unf* knockdown MB. Similar mechanisms have been shown in *C. elegans* and human. In human, the *unf* ortholog *PNR* is specifically expressed in rod photoreceptor cells to promote the rod-cell identity by repressing the expression of S-cone cell specific genes [11], [58]. Mutations in *PNR* leads to enhanced S-cone syndrome (ESCS) which is an inherited disease causing hypersensitivity to short-wave light due to increased numbers of S-cone cells at the expense of rod photoreceptor cells [11], [58]. However, in the present study, we did not observe any reciprocal cell number change in *unf* knockdown MBs. Thus, unlike *PNR* in human, *unf* in flies is not used to repress a default cell fate. Given that *unf* is required for the proper gene expression in all three major types of MB neurons, it may play a general role in assisting the temporal identity factors, such as *chinmo*, to diversify neuronal cell fates [3], [44].

Loss of proper identity might underlie the axon guidance defect observed in the *unf* knockdown MB. The *C. elegans* ortholog of *unf*, *fax-1*, has been suggested to regulate cell identities of 18 neurons, including both motor- and interneurons; in *fax-1* mutant, some neurotransmitters and synaptic proteins are not properly expressed in the neurons normally expressing *fax-1* [22], [59], [65]. Notably, like *unf* in fly MB neurons, *fax-1* is also required for axonal pathfinding for several *C. elegans* neurons, indicating a functional conservation among *unf* orthologs in different species [22], [59], [65].

*unf* has been hypothesized to act as a transcriptional repressor, because its ligand-binding domain (LDB) failed to activate gene expression [66]. The function of the human ortholog *PNR* to repress S-corn specific genes supports this hypothesis [58]. Contrary to the human *PNR*, in both flies and worms, loss of *unf* or *fax-1* leads to the down-regulation of many neural genes [22], [59]. However, since there is no evidence for gene activation by direct binding of *unf* or *fax-1*, it is possible that *unf* and *fax-1* regulate these neural genes indirectly by repressing other repressors.

In the MBs, *unf* primarily governs axonal morphogenesis during later larval and pupal development when different MB neuron types need to make distinct projections. For instance, in early pupae, α/β neurons undergo de-novo axonogenesis to form the α/β lobes while γ axons regenerate to make up the adult-specific γ lobe. Given the notion that *unf* promotes MB axonogenesis possibly through regulating neuron subtype identity, its selective involvement in late MB morphogenesis could simply reflect the importance of neuron subtype identity in ensuring diverse subtype-specific axonal morphogenesis. However, the larval γ neurons of *unf* knockdown MBs show normal cell fate as evidenced by proper expression of EcR-B1. This argues for a stage-specific function of *unf* in MB development. Notably, despite its stage-specific requirement, *unf* is enriched in the MBs through different developmental stages and into the adult, raising the possibility that its dynamic activity is patterned through temporal control of ligand availability. Recently, an *in vitro* study suggested that UNF is a heme binding protein [48]. Given the known relationship between heme and lipid metabolism [6], [12], [67], [68], heme levels can serve as an indicator for energy resource and developmental progress. Perhaps by detecting heme levels, *unf* may potentially coordinate the timing of the *unf-*medicated adult-specific cell fate determination and axonogenesis. However, *in vivo* evidence for the interaction between heme and *unf* remains lacking.

In conclusion, patterned NR activities govern various temporally regulated neural developmental processes of interest. UNF and its orthologs probably promote subtype neuronal differentiation in temporally controlled manners. Elucidating the regulation of NR activities and their control of neuron subtype identity should shed additional light on how diverse neuron types undergo differential morphogenesis and acquire different subtype-specific projections to construct the complex brain.

## Materials and Methods

### Generation of miRNA Fly Lines

The *UAS-miRNA* constructs were generated as described in the previous study [46]. The miRNA target sequences of 18 NR genes were shown in Table S1. The transgenic flies were generated by inserting *UAS-miRNA* constructs into the attp-16 site on the second chromosome using the integration system as previous described [69].

### Fly Strains

Beside the *UAS-miRNA* transgenic fly lines, the fly strains used in this study includes: (1) *asense-GAL4* [44]; (2) *GAL4-OK107* [51]; (3) *GAL4-MB247* [62]; (4) *GAL4-201Y* [53]; (5) *GAL4-NP21* [63]; (6) *GAL4-c305a* [64]; (7) *GAL4-c708* [44]; (8) *GAL4-c739* [53]; (9) *tubp-GAL80[ts];tubp-GAL80[ts],UAS-mCD8::GFP;OK107* [70]; (5) *Df(2R)ED2426/SM6a* (stock#9064, Bloomington stock center); (6) *hs-FLP,UAS-mCD8::GFP,FRTG13,tubp-GAL80/CyO;OK107*; (7) *hs-FLP[122];Sp/CyO;UAS>rCD2,y+>mCD8::GFP* [55], [56].

### Induction of Flip-Out Clones and MARCM Analysis

To generate flip-out clones, the newly hatched larvae with proper genotype were applied 30-minute heat-shock in 32°C water bath. For MARCM studies, larvae with proper genotype were collected in 2 hours after hatching, and cultured at the density of 100 larvae per food vial at 25°C. MARCM clones were induced by applying 1 hour heat-shock in 38°C water bath at various developmental stages.

### Immunohistochemistry and Confocal Microscopy

Fly brains were dissected, stained and mounted as described in our previous study (Lee and Luo, 1999). Primary antibodies used in this study include rabbit anti-UNF Ab (1∶4000); rat anti-mCD8 mAb (1∶100; Caltag), mouse anti-FasII mAb (1∶100; DSHB), mouse anti-Dac2-3 mAb (1∶200; DSHB), and rabbit anti-Trio Ab (1∶2000). FITC and Cy3 conjugated secondary antibodies (Jackson ImmunoResearch) were used at the dilution of 1∶200 and 1∶400, respectively. Immunofluorescent signals were collected by Zeiss LSM confocal microscope and processed using Osirix and Adobe Photoshop.

## Supporting Information

Table S1 (0.06 MB PDF)

## References

[1] Urbach R, Technau GM (2003). Segment polarity and DV patterning gene expression reveals segmental organization of the Drosophila brain.. Development.

[2] Pearson BJ, Doe CQ (2004). Specification of temporal identity in the developing nervous system.. Annu Rev Cell Dev Biol.

[3] Yu HH, Lee T (2007). Neuronal temporal identity in post-embryonic Drosophila brain.. Trends Neurosci.

[4] Luo L, O'Leary DD (2005). Axon retraction and degeneration in development and disease.. Annu Rev Neurosci.

[5] Truman JW (1990). Metamorphosis of the central nervous system of Drosophila.. J Neurobiol.

[6] Chawla A, Repa JJ, Evans RM, Mangelsdorf DJ (2001). Nuclear receptors and lipid physiology: opening the X-files.. Science.

[7] Francis GA, Fayard E, Picard F, Auwerx J (2003). Nuclear receptors and the control of metabolism.. Annu Rev Physiol.

[8] Kanai MI, Okabe M, Hiromi Y (2005). seven-up Controls switching of transcription factors that specify temporal identities of Drosophila neuroblasts.. Dev Cell.

[9] Kramer S, West S, Hiromi Y (1995). Cell fate control in the Drosophila retina by the orphan receptor seven-up: its role in the decisions mediated by the ras signaling pathway.. Development.

[10] Luo G, Chen Y, Li X, Liu T, Le W (2008). Nr4a2 is essential for the differentiation of dopaminergic neurons during zebrafish embryogenesis.. Molecular and Cellular Neuroscience.

[11] Milam AH, Rose L, Cideciyan AV, Barakat MR, Tang WX (2002). The nuclear receptor NR2E3 plays a role in human retinal photoreceptor differentiation and degeneration.. Proc Natl Acad Sci USA.

[12] Pardee K, Reinking J, Krause H (2004). Nuclear hormone receptors, metabolism, and aging: what goes around comes around.Transcription factors link lipid metabolism and aging-related processes..

[13] Tang LS, Alger HM, Pereira FA (2006). COUP-TFI controls Notch regulation of hair cell and support cell differentiation.. Development.

[14] Tran PV, Lee MB, Marín O, Xu B, Jones KR (2003). Requirement of the orphan nuclear receptor SF-1 in terminal differentiation of ventromedial hypothalamic neurons.. Mol Cell Neurosci.

[15] Zetterström RH, Solomin L, Jansson L, Hoffer BJ, Olson L (1997). Dopamine Neuron Agenesis in Nurr1-Deficient Mice.. Science.

[16] Zhou C (1999). The Nuclear Orphan Receptor COUP-TFI Is Required for Differentiation of Subplate Neurons and Guidance of Thalamocortical Axons.. Neuron.

[17] Kurusu M, Maruyama Y, Adachi Y, Okabe M, Suzuki E (2009). A conserved nuclear receptor, Tailless, is required for efficient proliferation and prolonged maintenance of mushroom body progenitors in the Drosophila brain.. Dev Biol.

[18] Liu H, Belz T, Bock D, Takacs A, Wu H (2008). The nuclear receptor tailless is required for neurogenesis in the adult subventricular zone.. Genes & Development.

[19] Shi Y, Shi Y, Lie DC, Lie DC, Taupin P (2004). Expression and function of orphan nuclear receptor TLX in adult neural stem cells.. Nature.

[20] Robinow S, Talbot WS, Hogness DS, Truman JW (1993). Programmed cell death in the Drosophila CNS is ecdysone-regulated and coupled with a specific ecdysone receptor isoform.. Development.

[21] Armentano M, Filosa A, Andolfi G, Studer M (2006). COUP-TFI is required for the formation of commissural projections in the forebrain by regulating axonal growth.. Development.

[22] Much JW, Slade DJ, Klampert K, Garriga G, Wightman B (2000). The fax-1 nuclear hormone receptor regulates axon pathfinding and neurotransmitter expression.. Development.

[23] Ponnio T, Conneely OM (2004). nor-1 Regulates Hippocampal Axon Guidance, Pyramidal Cell Survival, and Seizure Susceptibility.. Mol Cell Biol.

[24] Lee T, Marticke S, Sung C, Robinow S, Luo L (2000). Cell-autonomous requirement of the USP/EcR-B ecdysone receptor for mushroom body neuronal remodeling in Drosophila.. Neuron.

[25] Truman JW (1996). Steroid receptors and nervous system metamorphosis in insects.. Dev Neurosci.

[26] Chaturvedi RK, Beal MF (2008). PPAR: a therapeutic target in Parkinson's disease.. J Neurochem.

[27] Chu Y, Le W, Kompoliti K, Jankovic J, Mufson EJ (2006). Nurr1 in Parkinson's disease and related disorders.. J Comp Neurol.

[28] Culman J, Zhao Y, Gohlke P, Herdegen T (2007). PPAR-γ: therapeutic target for ischemic stroke.. Trends in Pharmacological Sciences.

[29] Jankovic J, Chen S, Le W (2005). The role of Nurr1 in the development of dopaminergic neurons and Parkinson's disease.. Prog Neurobiol.

[30] Le W, Le W, Xu P, Xu P, Jankovic J (2002). Mutations in NR4A2 associated with familial Parkinson disease.. Nat Genet.

[31] Le W, Pan T, Huang M, Xu P, Xie W (2008). Decreased NURR1 gene expression in patients with Parkinson's disease.. Journal of the Neurological Sciences.

[32] Serra H, Duvick L, Zu T, Carlson K, Stevens S (2006). RORα-Mediated Purkinje Cell Development Determines Disease Severity in Adult SCA1 Mice.. Cell.

[33] King-Jones K, Thummel CS (2005). Nuclear receptors–a perspective from Drosophila.. Nat Rev Genet.

[34] Maurange C, Cheng L, Gould AP (2008). Temporal transcription factors and their targets schedule the end of neural proliferation in Drosophila.. Cell.

[35] Schubiger M, Wade AA, Carney GE, Truman JW, Bender M (1998). Drosophila EcR-B ecdysone receptor isoforms are required for larval molting and for neuron remodeling during metamorphosis.. Development.

[36] Schubiger M, Truman JW (2000). The RXR ortholog USP suppresses early metamorphic processes in Drosophila in the absence of ecdysteroids.. Development.

[37] Schubiger M, Carre C, Antoniewski C, Truman JW (2005). Ligand-dependent de-repression via EcR/USP acts as a gate to coordinate the differentiation of sensory neurons in the Drosophila wing.. Development.

[38] Choi YJ, Lee G, Park JH (2006). Programmed cell death mechanisms of identifiable peptidergic neurons in Drosophila melanogaster.. Development.

[39] Brown HL, Truman JW (2009). Fine-tuning of secondary arbor development: the effects of the ecdysone receptor on the adult neuronal lineages of the Drosophila thoracic CNS.. Development.

[40] Wang J, Ma X, Yang JS, Zheng X, Zugates CT (2004). Transmembrane/juxtamembrane domain-dependent Dscam distribution and function during mushroom body neuronal morphogenesis.. Neuron.

[41] Boyle M, Nighorn A, Thomas JB (2006). Drosophila Eph receptor guides specific axon branches of mushroom body neurons.. Development.

[42] Ng J, Nardine T, Harms M, Tzu J, Goldstein A (2002). Rac GTPases control axon growth, guidance and branching.. Nature.

[43] Lee T, Luo L, Lee A (1999). Development of the Drosophila mushroom bodies: sequential generation of three distinct types of neurons from a neuroblast.. Development.

[44] Zhu S, Lin S, Kao CF, Awasaki T, Chiang AS (2006). Gradients of the Drosophila Chinmo BTB-zinc finger protein govern neuronal temporal identity.. Cell.

[45] Awasaki T, Ito K (2004). Engulfing action of glial cells is required for programmed axon pruning during Drosophila metamorphosis.. Curr Biol.

[46] Chen CH, Huang H, Ward CM, Su JT, Schaeffer LV (2007). A synthetic maternal-effect selfish genetic element drives population replacement in Drosophila.. Science.

[47] Shi L, Yu HH, Yang JS, Lee T (2007). Specific Drosophila Dscam juxtamembrane variants control dendritic elaboration and axonal arborization.. J Neurosci.

[48] de Rosny E, de Groot A, Jullian-Binard C, Borel F, Suarez C (2008). DHR51, the Drosophila melanogaster Homologue of the Human Photoreceptor Cell-Specific Nuclear Receptor, Is a Thiolate Heme-Binding Protein.. Biochemistry.

[49] Sung C, Wong L, Chang Sen L, Nguyen E, Lazaga N (2009). The unfulfilled/DHR51 gene of Drosophila melanogaster modulates wing expansion and fertility.. Dev Dyn.

[50] Awasaki T, Saito M, Sone M, Suzuki E, Sakai R (2000). The Drosophila trio plays an essential role in patterning of axons by regulating their directional extension.. Neuron.

[51] Connolly JB, Roberts IJ, Armstrong JD, Kaiser K, Forte M (1996). Associative learning disrupted by impaired Gs signaling in Drosophila mushroom bodies.. Science.

[52] Crittenden JR, Skoulakis EM, Han KA, Kalderon D, Davis RL (1998). Tripartite mushroom body architecture revealed by antigenic markers.. Learn Mem.

[53] Yang MY, Armstrong JD, Vilinsky I, Strausfeld NJ, Kaiser K (1995). Subdivision of the Drosophila mushroom bodies by enhancer-trap expression patterns.. Neuron.

[54] McGuire SE, Le PT, Osborn AJ, Matsumoto K, Davis RL (2003). Spatiotemporal rescue of memory dysfunction in Drosophila.. Science.

[55] Wong AM, Wang JW, Axel R (2002). Spatial representation of the glomerular map in the Drosophila protocerebrum.. Cell.

[56] Zhu S, Chiang AS, Lee T (2003). Development of the Drosophila mushroom bodies: elaboration, remodeling and spatial organization of dendrites in the calyx.. Development.

[57] Lee T, Luo L (1999). Mosaic analysis with a repressible cell marker for studies of gene function in neuronal morphogenesis.. Neuron.

[58] Chen J, Rattner A, Nathans J (2005). The rod photoreceptor-specific nuclear receptor Nr2e3 represses transcription of multiple cone-specific genes.. J Neurosci.

[59] Wightman B, Ebert B, Carmean N, Weber K, Clever S (2005). The C. elegans nuclear receptor gene fax-1 and homeobox gene unc-42 coordinate interneuron identity by regulating the expression of glutamate receptor subunits and other neuron-specific genes.. Dev Biol.

[60] Martini SR, Davis RL (2005). The dachshund gene is required for the proper guidance and branching of mushroom body axons in Drosophila melanogaster.. J Neurobiol.

[61] Martini SR, Roman G, Meuser S, Mardon G, Davis RL (2000). The retinal determination gene, dachshund, is required for mushroom body cell differentiation.. Development.

[62] McGuire SE, Le PT, Davis RL (2001). The role of Drosophila mushroom body signaling in olfactory memory.. Science.

[63] Tanaka NK, Awasaki T, Shimada T, Ito K (2004). Integration of chemosensory pathways in the Drosophila second-order olfactory centers.. Curr Biol.

[64] Krashes MJ, Keene AC, Leung B, Armstrong JD, Waddell S (2007). Sequential use of mushroom body neuron subsets during drosophila odor memory processing.. Neuron.

[65] Wightman B, Baran R, Garriga G (1997). Genes that guide growth cones along the C. elegans ventral nerve cord.. Development.

[66] Palanker L, Necakov AS, Sampson HM, Ni R, Hu C (2006). Dynamic regulation of Drosophila nuclear receptor activity in vivo.. Development.

[67] Bauer M, Hamm AC, Bonaus M, Jacob A, Jaekel J (2004). Starvation response in mouse liver shows strong correlation with life-span-prolonging processes.. Physiol Genomics.

[68] Lorenz RT, Parks LW (1991). Involvement of heme components in sterol metabolism of Saccharomyces cerevisiae.. Lipids.

[69] Markstein M, Pitsouli C, Villalta C, Celniker S, Perrimon N (2008). Exploiting position effects and the gypsy retrovirus insulator to engineer precisely expressed transgenes.. Nat Genet.

[70] Shi L, Lin S, Grinberg Y, Beck Y, Grozinger C (2007). Roles of Drosophila Kruppel-homolog 1 in neuronal morphogenesis.. Devel Neurobio.

[71] Honjo K, Furukubo-Tokunaga K (2009). Distinctive neuronal networks and biochemical pathways for appetitive and aversive memory in Drosophila larvae.. J Neurosci.

[72] Marin EC, Watts RJ, Tanaka NK, Ito K, Luo L (2005). Developmentally programmed remodeling of the Drosophila olfactory circuit.. Development.

[73] Honjo K, Furukubo-Tokunaga K (2005). Induction of cAMP response element-binding protein-dependent medium-term memory by appetitive gustatory reinforcement in Drosophila larvae.. J Neurosci.

