# Supplementary material for: Nuclear Receptor Unfulfilled Regulates Axonal Guidance and Cell Identity of Drosophila Mushroom Body Neurons
Source: PLoS One. 2009 Dec 22;4(12):e8392. doi: 10.1371/journal.pone.0008392 (PMC2793019; doi:10.1371/journal.pone.0008392)
Supplement: Table S1 — (0.06 MB PDF) [file pone.0008392.s001.pdf]

Table S1. Complete survey of 18 nuclear receptors' function in mushroom body neurogenesis by miRNA knockdown.

Gene name	Subfamily	miRNA transgene integration site	OK107>miRNA phenotype	Documented function in MB	miRNA target sequences
**EcR**	1H	attp-16	Small eye. 100% (n=12) MB had pruning defect	γ pruning	TATGAGCAGCCATCTGAAGAGG<br>CATTGAAGACCTGCTGCATTTC
**ERR**	3B	attp-16	normal	no	TCAAACTCTTACCAGACTATGC<br>CCACTTAACGACCAGATGAAGC
**E78**	1D/E	attp-16	normal	no	GGTTGCAATAGTGCCAATAACA<br>AGATCAGATACACGTCTACTCA
**FTZ-F1**	5A	attp-16	30% (n=50) MB contained ectopic FasII-positive bundles.	no	TGTAGTCCCTGATAAAGCAATT<br>CGTACTTTTAGCGTCCTACATA
**HNF4**	2A	attp-16	normal	no	GGTTCTGCAGTCTATTACCTGG<br>CATGCCCGTCAAGCACTACAAT
**DHR3**	1F	attp-16	normal	no	ACACTAGCTTACATAGAACTGG<br>GCAGAACCTATAAACTGATAAG
**DHR38**	4A	attp-16	normal	no	TCACCACCCATCTCGTTGATCA<br>CAAGCGTATCATCACAACTTGC
**DHR96**	1I/J	attp-16	normal	no	GCATTTAATGCCTATGTTGATG<br>CGAGAACATAATCCTGATCATG
**DHR39**	5A	attp-16	normal	no	CGACACCTCTCAAACTTTTACA<br>CAACTCACCAATCCACTACTGG
**DHR4**	6A	attp-16	normal	no	AGTGGCACGAGATCCTTATCCT<br>TCTCCCACATACCAGAGATCCA
**DHR78**	2C/D	attp-16	normal	no	GGTTGAGACGTTCATCAAAAGC<br>GATGTGCAGTGTGCCTTTCATG
**DHR83**	2E	attp-16	normal	no	CGTCCTGGAACTCAACTTTATG<br>ACTGCGCGCTTTCTTGTATGTT
**UNF**	2E	attp-16	100% (n > 50) MB lobes stopped shortly at the end of peduncle	no	TCATCGATGACCCGGATTAAGG<br>ACACCCATCAACACGAAACAGT<br>CCAGTCCCACCATGGAAAATGA#
**DSF**	2E	attp-16	normal	no	TCTGCAATCCAGCTCTAAAACG<br>GGTTCAAATACTTCCATCAACG
**E75**	1D/E	attp-16	pupal lethal. larval MB was normal	no	CATCTGAAGCGACAGATTGTGG<br>GCAGCGATATTATCCAAAACTA
**SVP**	2F	attp-16	normal	no	GCTGTCATGCAGCAAACATACG<br>GTAACCATTACGAGTAAAAACA
**TLL**	2E	attp-16	100% (n=12) MB had only a few neurons left	efficient proliferation	TGTGCCATCTGAACATTGACAG<br>GCATCGAGTCGCATTCTATACC
**USP**	2B	attp-16	100% (n=12) MB had pruning defect	γ pruning	CATCTTCGACCGCATATTGTCG<br>TGATGACTTCATGACCAATAGC

Subfamily designation is as described by Ruau, D. et al. Update of NUREBASE: nuclear hormone receptor functional genomics. *Nucleic Acid Res.* 32 (database issue), D165-D167 (2004). #Target sequence used for the second *DHR51* miRNA (miRNA-b).
